# Low-Cost Otolaryngology Simulation Models for Early-Stage Trainees: A Scoping Review

**DOI:** 10.21203/rs.3.rs-3673832/v1

**Published:** 2023-12-08

**Authors:** Joselyne Nzisabira, Sarah Nuss, Estephanía Candelo, Ernest Aben Oumo, Keshav V. Shah, Eric K. Kim, Joshua Wiedermann, Ornella Masimbi, Natnael Shimelash, Mary Jue Xu

**Affiliations:** University of Global Health Equity; Brown University Warren Alpert Medical School; Fundación Valle del Lili; University of Global Health Equity; University of Pennsylvania; University of California, San Francisco; Mayo Clinic; University of Global Health Equity; University of Global Health Equity; University of California, San Francisco

**Keywords:** Low-cost, simulation, otolaryngology, low- and middle-income country, task trainer, medical student, trainee

## Abstract

**Importance:**

There is a notable lack of low-cost OHNS simulation models that are relevant for early medical trainees and students. By conducting this study, we will understand the current landscape of low-cost otolaryngology-head and neck surgery simulation for early medical trainees and students.

**Objectives:**

Medical simulation is essential for surgical training yet is often too expensive and inaccessible in low- and middle-income countries (LMICs). Furthermore, in otolaryngology-head and neck surgery (OHNS), while simulation training is often focused on senior residents and specialists, there is a critical need to target general practitioners who carry a significant load of OHNS care in countries with limited OHNS providers. This scoping review aims to describe affordable, effective OHNS simulation models for early-stage trainees and non-OHNS specialists in resource-limited settings and discuss gaps in the literature.

**Evidence Review:**

This scoping review followed the five stages of Arksey and O’Malley’s Scoping Review Methodology. Seven databases were used to search for articles. Included articles discussed physical models of the ear, nose, or throat described as “low-cost,” “cost-effective,” or defined as <$150 if explicitly stated; related to the management of common and emergent OHNS conditions; and geared towards undergraduate students, medical, dental, or nursing students, and/or early-level residents.

**Findings:**

Of the 1706 studies screened, 17 met the inclusion criteria. Most studies were conducted in HICs. Most models were low fidelity (less anatomically realistic) models. The most common simulated skills were peritonsillar abscess aspiration and cricothyrotomy. Information on cost was limited, and locally sourced materials were infrequently mentioned. Simulations were evaluated using questionnaires and direct observation.

**Conclusion and Relevance:**

Low-cost simulation models can be beneficial for early medical trainees and students in LMICs, addressing resource constraints and improving skill acquisition. However, there is a notable lack of contextually relevant, locally developed, and cost-effective models. This study summarizes existing low-cost OHNS simulation models for early-stage trainees and highlights the need for additional locally sourced models. Further research is needed to assess the effectiveness and sustainability of these models.

## Introduction

Medical simulation is a valuable component of training.^1^ Historically, simulation usage has been predominantly centered in high-income countries (HICs). Consequently, there exists an opportunity to expand access to simulation education in low- and middle-income countries (LMICs).^2,3^ While low-cost simulation models have been explored in HICs, the specific models used in these settings may not be applicable to LMICs due to lacking the same resources. Studies have demonstrated that using locally sourced materials and readily available devices is cost-effective.^4^ Furthermore, low-fidelity, or less anatomically realistic, simulation may confer similar benefits compared to high-fidelity, or highly anatomically realistic, simulation though with lower costs.^5,6^ Despite the potential benefits of simulation in LMICs, there is limited literature, particularly for surgical specialties where workforce shortages, ethical considerations, and financial constraints limit opportunities for practice.^7^

In otolaryngology-head and neck surgery (OHNS), simulation training has an opportunity to address the burden of disease centered in LMICs through training of general practitioners (GPs) and primary care providers in regions where subspecialists are limited. The burden of OHNS disease is high, with 1.5 billion people worldwide experiencing hearing loss, primarily in LMICs.^8^ Paradoxically, low-income countries have 50 times fewer OHNS providers than high income countries.^9^ Given the burden of OHNS disease far outweighs the current number of providers, it is imperative to train primary healthcare provider to help increase access to essential OHNS care. Simulation is a central component of many HIC OHNS training programs, ^10–13^,^14^; however, many models are largely directed at the skill set of senior residents and physicians. Given that primary care providers such as GPs in LMICs may be the first or only providers available in rural or first-level hospitals, the opportunity to develop skills that are critical for managing OHNS emergencies and common conditions is essential to developing confidence and preventing morbidity and mortality.

To address the gap in simulation models for primary care practitioners in common and emergent OHNS conditions, this scoping review aims to describe and evaluate available low-cost OHNS simulation models geared toward early-stage medical trainees or GPs.

## Methods

### Study Design

Given limited and heterogenous literature, a scoping review was selected and conducted in February 2023 in accordance with Arksey and O’Malley’s Scoping Review Methodology and following the PRISMA (Preferred Reporting Items for Systematic Reviews and Meta-Analyses) Extension for Scoping Reviews Guidelines.^15,16^ The search strategy aimed to address the research question regarding the outcomes of using low-cost OHNS simulation models for early-stage trainees in education.

### Literature Search

A search strategy was developed to capture the maximal results, which included the main search concepts of “simulation,” “otolaryngology,” “education,” and “low cost.” These terms were combined using Boolean operators OR (within critical constructed concepts) and AND (between key concepts). The specific search strategy was adapted to each data base. The search was conducted in the following databases: PubMed, MEDLINE, EBSCO, Scopus, Science Direct, CINAHL, EMBASE, and Web of Science (Supplemental Table 1).

Inclusion criteria included studies of any language that discussed the development or implementation of a physical model of the ear, nose, or throat that were explicitly described as “low-cost,” “cost-effective,” or defined as <$150 if explicitly stated related to the care or management of OHNS conditions (operative or non-operative). Models were only considered if they were applicable for training of undergraduate students, medical, dental, or nursing students, and/or early-level residents, and we excluded simulations that would not be applicable to a GP (i.e., advanced OHNS resident level skills). Original research of any study type was included. Letters to the editor, abstracts, systematic reviews, virtual reality simulations, electronic simulations, and studies that utilized mannequin models were not included.

The study team completed a primary title and abstract screening using a Covidence database (Veritas Health Innovation Ltd, Melbourne) based on the search criteria. Two reviewers each independently screened the titles and abstracts of all identified articles for relevance to the research question. A third independent reviewer resolved disagreements over article eligibility. In the full-text review, data was extracted and recorded following the Arksey and O’Malley’s “descriptive-analytical” approach for data extraction, and the information was summarized from selected articles on an Excel spreadsheet.^15^ At least two authors reviewed extracted data from the included articles. A third reviewer resoled any remaining conflicts. Snowball sampling was used to identify gray literature from study reference lists.

### Statistical analysis

Outcomes included study characteristics (authors, year, language, journal of publication, study design), context (study country, target population) simulation details (specialty of simulation model, cost, fidelity of model, materials used, local sourcing of materials, condition being simulated), and model evaluation (evaluation of surgical skill and efficacy of model). Summary statistics were performed using Microsoft Excel. Categorical variables were presented as counts and percentages n(%). There were no continuous data.

### Results

**Figure 1:**
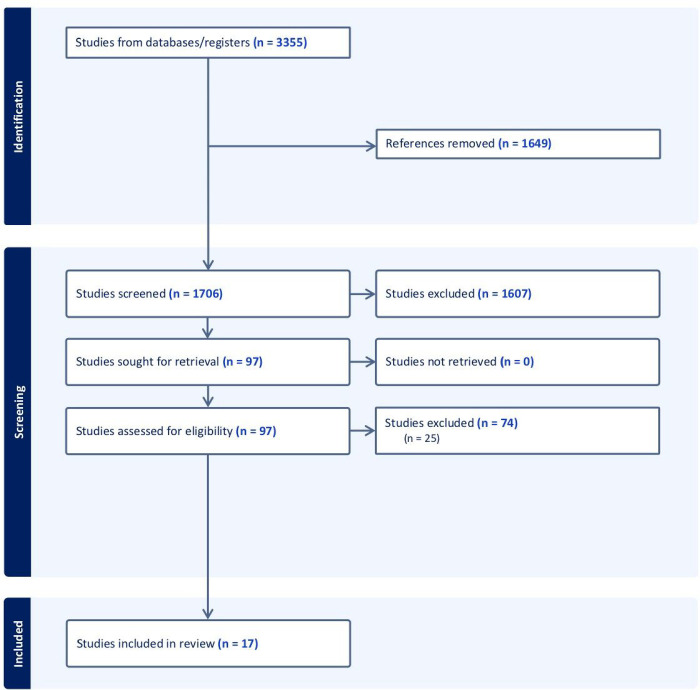
PRISMA Flow diagram of data analysis procedure.

The initial search returned 3355 studies. After 1649 duplicates were removed, 1706 studies underwent title and abstract screening. Of these, 1607 were excluded. Ninety-nine studies were screened for full text review based on inclusion and exclusion criteria. Seventeen studies met inclusion criteria (Fig. 1). Table 1 provides an overview of the included low-cost simulation models for essential OHNS conditions.

### Characteristics of studies

**Figure 2:**
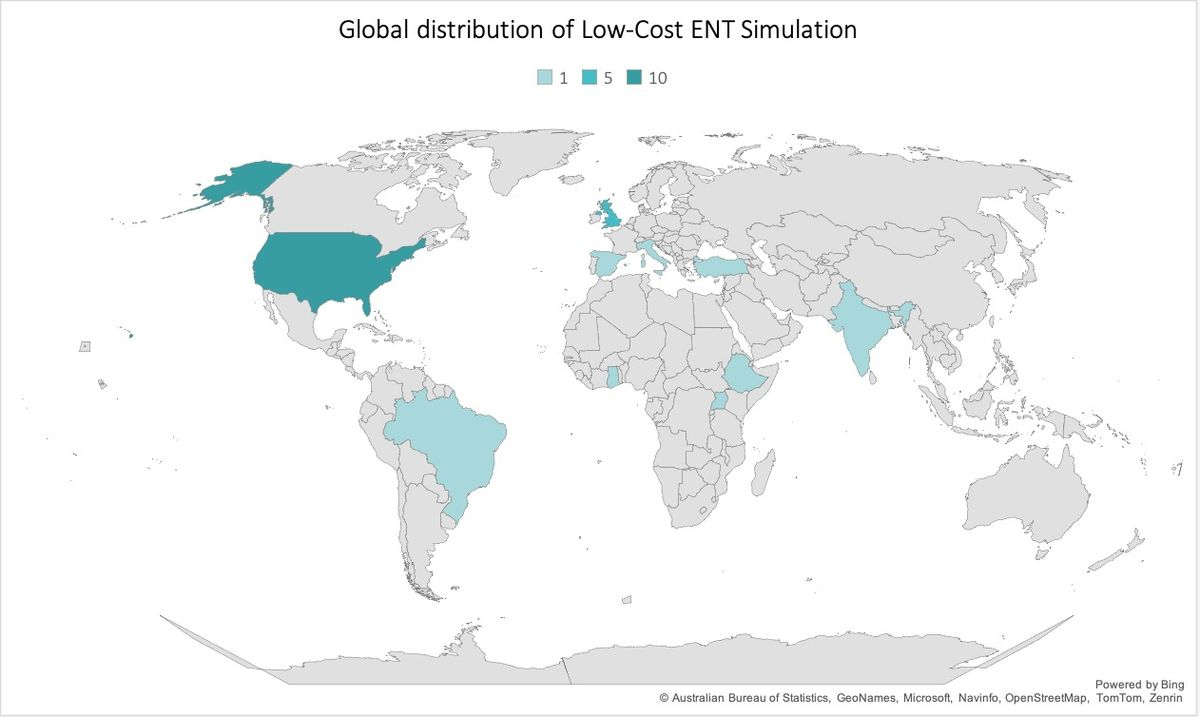
Global distribution of Low-Cost ENT Simulation Model Studies

Of the studies examined, 82% (n = 14) of studies were conducted in HICs, and the majority were conducted in the United States or in the United Kingdom (Fig. 2). 94% (n = 16) of the studies utilized a cross sectional study design. Most articles targeted general OHNS care (n = 8, 47%). 82% (n = 14) of the models were low-fidelity models (less anatomically realistic), which was defined by the primary study authors. The characteristics of the studies are summarized in Table 2.

### Skills

The most common simulated skills were peritonsillar abscess aspiration (n = 6, 35%), cricothyrotomy (n = 4, 24%), myringotomy with tube placement (n = 2, 12%), and other ear models (2, 12%). Nasal packing (n = 1, 6%), auricular hematoma (n = 1, 6%), and tracheostomy care (n = 1, 6%) were also included.

### Audience

One (6%) study was geared towards medical students, eight (47%) towards residents, two (12%) towards both medical students and residents, one (6%) towards nurses, one towards anesthesia students, and one (6%) towards paramedics. Out of the eight resident-focused models, three were geared towards emergency medicine residents. Two (12%) models were geared towards attendings or consultants, and both models were included given the models’ transferability to simulate other more basic skills.

### Cost

Eleven (65%) models reported a dollar value associated with their model. The average price per model was $36.45 USD (range: $10 – $105). The remaining models were described as “low-cost” by authors without specific information about the cost of the materials. Fifteen (88%) studies reported using locally sourced materials.

### Simulation Evaluation

Sixteen (94%) studies assessed model efficacy. Models were evaluated using both questionnaires (n = 8, 47%), direct observation of skills (n = 4, 24%), or both (n = 4, 24%). Three of the eight studies that included direct observation (38%) used video monitoring to evaluate clinical skill. Participant questionnaires included a variety of themes such as participants’ comfort with the skill, model realism, ease of use, and participant confidence performing the skill.

## Discussion

Given the substantial burden of OHNS disease worldwide and current limited OHNS workforce, simulation training tools tailored for primary care providers are critical in developing OHNS knowledge and skills to increase access to OHNS care globally.^8,9^ Existing low-cost OHNS simulations primarily target residents and consultants and can often overlook the essential skill set required by GPs.^17,18^ These skills encompass emergent and common OHNS conditions such as epistaxis, emergent surgical airway, and ear and nose foreign body removal. Equipping medical students and early-trainees with basic OHNS care skills is vital. This type of task shifting can alleviate delays in care, transportation challenges, and alleviate the burden on tertiary centers.

This is the first study to evaluate low-cost OHNS simulations tailored to GPs and early-trainee education, emphasizing locally sourced models. The low number of studies highlights that simulations addressing the skill set of early trainees and primary care providers is an area for future educational research depending on regional needs and resource availability. Our findings describe the available low-cost simulations in OHNS and highlights insufficient availability of such models. Future work should focus on developing additional low-cost, contextually appropriate models to bridge gaps in healthcare training and delivery in resource-constrained settings.

Simulated medical models have proven highly effective in imparting essential OHNS procedure skills and can provide an important avenue to improve surgical training in resource constrained environments. However, our data show that most low-cost simulation models (n = 14, 82%) are developed and utilized in HIC, which aligns with prior studies that report a lack of locally developed low-cost simulations in LMIC contexts.^7^ Furthermore, many “low-cost” simulation models rely on high-cost materials such as 3D printers or specialized mannequins, which may not be available in LMICs. When considering model sustainability and applicability of these models in LMICs, it is important to recognize the limitations of certain high-fidelity models in such resource-constrained environments. Prior studies demonstrate that low fidelity simulation models do not necessarily lead to worse skill outcomes, which emphasizes the potential of low-cost, less intricate models as valuable tools for skill acquisition.^5,19^

A previous systematic review of low-cost simulations in OHNS identified 18 studies on low-cost ENT simulations.^14^ However, only five of these simulations were relevant to GPs as shown in Table 3. In contrast, our study included 17 simulations directly applicable to GPs. There is potential for expanding the range, reach, and applications of existing models. Most of the models in our study focused on peritonsillar abscess simulations, which may not always fall within a GP’s scope of practice. These results highlight the need to develop additional models for skills like epistaxis management and nasal/ear foreign body removal. Additionally, several of the existing models could be adapted for a broader set of GP-level skills, such as using ear models for foreign body removal and cerumen management, in addition to myringotomy. There is also a clear need for alternatives to animal models, which can be harder to procure or reuse, leading to higher operation and maintenance costs. Additionally, most models in this study did not explore the use of locally sourced materials. Collaborating with LMICs to adapt models to utilize locally available materials is an essential next step to enhance accessibility and effectiveness. Finally, our study identified heterogeneity in evaluations of the efficacy of these simulations in augmenting the knowledge, skills, and confidence of GPs. This suggests that future research should incorporate standardized metrics to assess the utility of low-cost OHNS simulations.

Our study has some limitations. Not all the studies we included provided exact cost information for the simulations, which, if available, could have contributed to our understanding of the cost-effectiveness of these models. We also did not independently evaluate fidelity and instead relied on fidelity assessments as reported by the authors for the scope of this study.. Furthermore, excluding studies involving 3D printing or mannequins might have resulted in overlooking potentially useful insights regarding the development and components of these models. As 3D printing technology becomes more affordable, cost and access may not be a barrier in the future, opening exciting possibilities for its integration into future research studies and innovations across various fields. Additionally, a notable portion of the studies reviewed did not compare efficacy directly to high-fidelity models, highlighting the need for further research regarding the effectiveness of these simulations.

## Conclusion

Low-cost, locally sourced OHNS simulations for GPs, early trainees, and students hold immense promise in LMICs. This tailored simulation-based training not only addresses the financial constraints faced by educational institutions but also considers local factors, including the local burden of OHNS diseases, available resources, hospital infrastructure, and the distinct roles and responsibilities of GPs in these settings. By conducting country-specific studies, these simulations could offer a practical and sustainable solution to enhance OHNS knowledge and skills among primary care providers, ultimately improving healthcare delivery and patient outcomes. Our scoping identified a range of potential simulation models that hold promise for replication in LMICs, along with crucial gaps that warrant exploration for the development of contextually relevant, low-cost models.

## Figures and Tables

**Table 1:** Summary of Included Articles

Study	Reference	Year	Country	OHNS Procedure	Cost	Materials
1	Alicja Chudek D, Wilson I, Hogg E, et al.	2021	UK	PTA Aspiration	“Low-Cost”	Latex glove, custard, balloon, paper cup, tape, jelly, green food coloring, resuscitation mask (optional; to orient trainee)
2	Bright RR, Varghese L, Kurien R	2021	India	Nasopharyngeal swabbing, endoscopy, anterior and posterior nasal packing	$10 USD	Thermoplastic ray cast, plaster of Paris, cardboard
3	Bunting H, Wilson BM, Malloy KM, Malekzadeh S	2015	USA	Needle aspiration and I&D	“Low-Cost”	Water balloon, lotion, food coloring, glue, paper cup, gelatin, cookie cutter, clay, manikin face mask, Styrofoam pool noodle, pencil
4	Chiesa Estomba, C. M.; Melendez Garcia, J. M.; Hamdam Zavarce, M. I.; Betances Reinoso, F. A	2015	Spain	Transtympanic grommet placement	“Low-Cost”	Wood, anchoring tape, foam, scissors, 5 mL syringes, wood glue and latex gloves
5	Aho, J. M.; Thiels, C. A.; AlJamal, Y. N.; Ruparel, R. K.; Rowse, P. G.; Heller, S. F.; Farley, D. R.	2015	USA	Cricothyrotomy	“Low-Cost”	Cardboard toilet paper roll, Styrofoam tubing, cardboard, zip tie, fabric
6	Patricia K T Pothier	2006	USA	Tracheostomy model for suctioning simulation	<$100 USD	Plastic pipe, tracheotomy tube, pink rubber ball, tape, white plastic (anchor the model), white Velcro, large corks (optional)
7	Taylor, S. R.; Chang, C. W.	2014	USA	PTA Aspiration	Low-cost version approximately $10 USD	Latex moulage, clay mold “negative space,” rubber band, 2.5 inch-diameter polyvinylchloride pipe, small balloon, polyurethane foam, abscess liquid (teaspoons sugar-free vanilla pudding powder with 30cc coffee creamer), rubber tongue from patient simulator mannequin
8	Washington, C.H., Tyler, F.J., Davis, J. et al.	2014	USA	PTA Aspiration	$10 USD	Latex moulage
9	Bhalla, S.; O’Byrne, L.; Beegun, I.; Amos, D.; Jones, J. A.; Awad, Z.; Tolley, N.	2021	UK	PTA Aspiration	< $11 USD	Cork, balloon, color gel soap, plastic inserts, and rubber
10	Clark, M. P. A.; Westerberg, B. D.; Mitchell, J. E.	2016	UK	Procedures in the ear canal	<$100 USD	Round bead, blunt-right angled hook, latex glove with a hole punched through, graft material was cigarette paper, sewing needle
11	Botto, F. S.; Ingrassia, P. L.; Donato, P.; Garzaro, M.; Aluffi, P.; Gentilli, S.; Olina, M.; Grossini, E.	2019	Italy	Cricothyrotomy, percutaneous and surgical tracheostomy	$25 USD	Wooden tablet, foam square, porcine trachea
12	Molin, N.; Chiu, J.; Liba, B.; Isaacson, G.	2020	USA	Myringotomy tube (MT) placement	“Low-Cost”	110 mm plastic coated, resin-core bocce ball with a single cylindrical working shaft and a paper tympanic membrane target on an angled plastic slide. A wooden bocce ball may also be used. The bocce ball rests on a plastic pedestal to allow rotation in all directions
13	Muckler, V. C.; Kampo, S.; Morgan, B.	2017	Ghana	Needle cricothyrotomy	$14 USD	Paper towel roll, 3.8-cm ribbed pencil grippers ($1.39 per 5-pack), adhesive tape ($2.35 for 2 rolls), Esmarch bandage, 3-mL syringe, 14- or 16-gauge angiocatheters or needles, optional small balloon, guidewire, and scissors
14	Ng, V.; Plitt, J.; Biffar, D.	2018	USA	PTA Aspiration	$105 USD	Discarded headskin, hardware cloth, PVC sewer and drain fitting, 4” outer diameter, NDS drain grate, 4” inner diameter, duct tape, zip ties, utility hook hangers & screws, scrap plywood, scrap foam or towels, Dimethicone barrier lotion, Water balloons (500 ct), 8oz paper ice cream cups (100 ct), Craft sticks (100 ct), Cyanoacrylate glue, 600oz ballistic gelatin, food coloring, cotton balls/pads, paint
15	Ozkaya Senuren, C.; Yaylaci, S.; Kayayurt, K.; Aldinc, H.; Gun, C.; Şimşek, P; Tatli, O.; Turkmen, S.	2020	Turkey	Cricothyroidotomy	$10 USD	Styrofoam, a sheep trachea, and a double layer of chicken skin. Sterile gloves, a scalpel, a scalpel handle, a hook, an endotracheal tube, sponge, syringe, and antiseptic solution were used in carrying out the procedure
16	Walsh, R.; Fennessy, T.; Pauw, E.; Lajeunesse, M.; Couperus, K.	2022	USA	Auricular hematoma repair	“Low-Cost”	Bell papers, cardboard, plastic wrap, ketchup
17	Rotimi, O.; Haymes, A.; Dodds, I.; Bhutta, M.	2022	UK	Otoscopic examination	Entire platform could cost < $13 USD (planned discount for LMICs)	Platform A (traditional otoscope and manikin ear simulator), Platform B (Tympahealth digital otoscope and manikin ear simulator), Platform C (traditional otoscope and SimEar simulator)

**Table 2:** Description of Included Articles by Type

		n (%)
Research design	Cross sectional study	16 (94.1%)
	Cohort	0 (0.0%)
	Randomized control trial	0 (0.0%)
	Other (mixed methods)	1 (5.9%)_
ENT specialty	Otology	5 (29.4%)
	Rhinology	1 (5.9%)
	Head and Neck Surgery	2 (11.8%)
	Facial Plastics	0 (0.0%)
	Laryngology	1 (5.9%)
	General ENT	8 (47.1%)
Fidelity of Model	Low	14 (82.4%)
	Low to medium	1 (5.9%)
	Medium	1 (5.9%)
	High	1 (5.9%)
World Bank Lending Status	High-Income Country	14 (82.4%)
	Upper-Middle Income Country	1 (5.9%)
	Lower-Middle Income Country	2 (11.8%)

**Table 3:** Low-cost OHNS simulations identified in prior studies versus our results

	Pankhania et al.	Our Study
Total Number of Studies	18	17
Studies Relevant to General Practitioners (GPs)	5 (28%)	17 (100%)
Specific Simulations Relevant to GPs	• Myringotomy Tubes: 4 • Peritonsillar Abscess: 1	• Peritonsillar Abscess: 6 • Cricothyrotomy: 4 • Mixed Ear Skills: 2 • Myringotomy: 2 • Auricular hematoma: 1 • Epistaxis: 1 • Tracheostomy Care: 1
Studies Not Related to GP Skills	13 (e.g., transcervical laryngeal injection, LTR, sinus surgery trainer, endoscopic ear surgery)	N/A
Evaluation of Usefulness (Knowledge, Skills, Confidence)	Assessed in 4 (22%) models	Assessed in 16 (94%) models
Use of Locally Sourced Materials	Not mentioned	Identified as an area for improvement
Geographic Diversity of Settings	Not mentioned	Identified as an area for improvement

## Data Availability

All data is previously published publically available literature that has undergone prior IRB review.

## Key Points

### Question:

What is the current landscape of low-cost otolaryngology-head and neck surgery simulation for early medical trainees and students?

### Finding:

In this scoping review we identified 17 studies that met inclusion criteria. Most studies were developed in high-income countries, and most models were not locally sourced.

### Meaning:

There is a notable lack of low-cost OHNS simulation models that are relevant for early medical trainees and students.
